# Supplementary material for: BRG1 and NPM-ALK Are Co-Regulated in Anaplastic Large-Cell Lymphoma; BRG1 Is a Potential Therapeutic Target in ALCL
Source: Cancers (Basel). 2021 Dec 29;14(1):151. doi: 10.3390/cancers14010151 (PMC8750310; doi:10.3390/cancers14010151)
Supplement: Supplementary file 1 [file cancers-14-00151-s001.zip › cancers-1526480-supplementary Figures.pdf]

cancers

MDPI

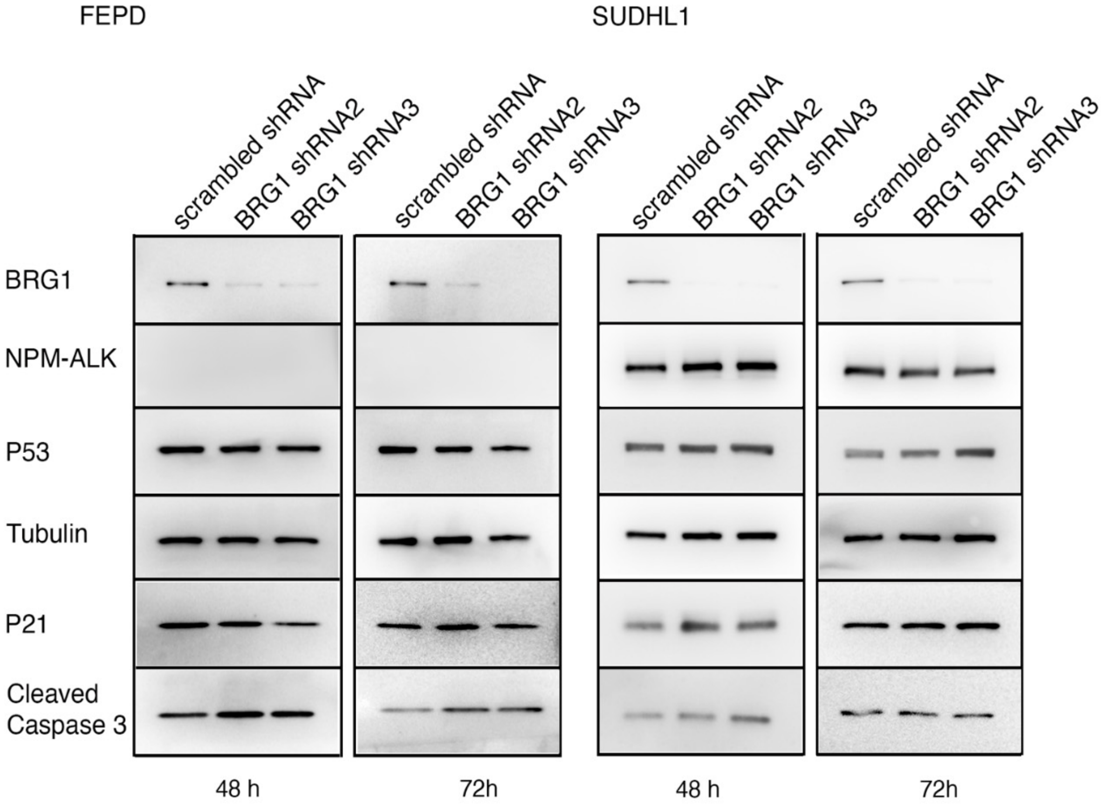

Figure S1. BRG1 knockdown with specific shRNA in ALCL the cell lines FEPD and SUDHL1 does not affect expression of key cell cycle regulatory proteins. Cell lysates of FEPD and SUDH1 cells were prepared at 48 and 72 hours post puromycin selection following transfection with scrambled shRNA, or BRG1 shRNA2 or shRNA3 and probed by Western blot for the indicated proteins. Data are representative of biological triplicates.

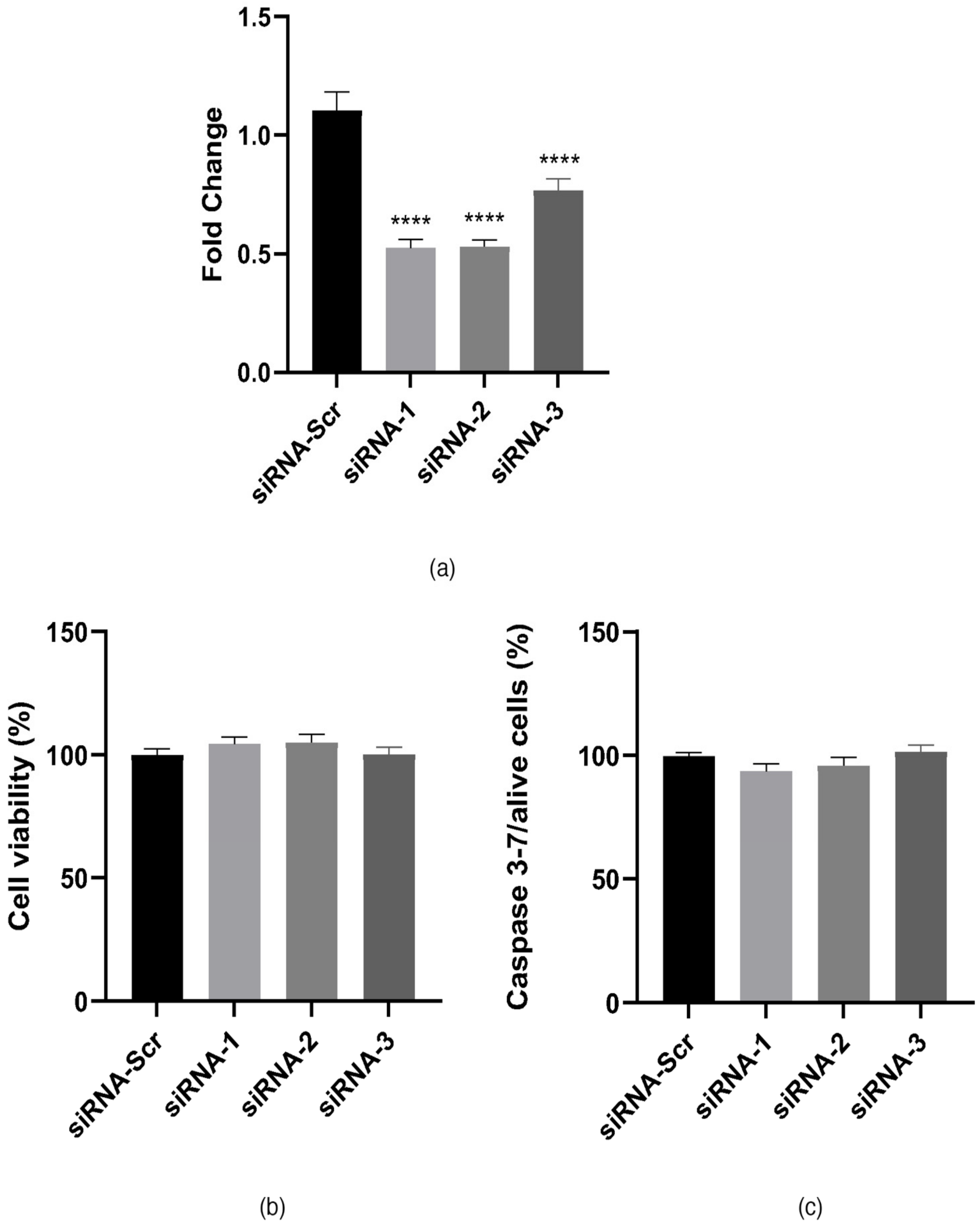

Figure S2. BRG1 knockdown does not affect cell viability nor apoptosis of murine CD4/NPM-ALK derived tumour cells. BRG1 transcript levels are significantly deceased following transfection of the cells with mouse-specific BRG1 siRNA. (**a**) but this does not affect cell viability (**b**) nor caspase 3/7 activity (**c**) 6 days after transfection. Results are shown as means ± SEM from three independent experiments. One-way ANOVA with Tukey's post-test has been used for statistical comparison; ****$p < 0.0001$. Scr: scrambled.
