# Supplementary material for: BRG1 and NPM-ALK Are Co-Regulated in Anaplastic Large-Cell Lymphoma; BRG1 Is a Potential Therapeutic Target in ALCL
Source: Cancers (Basel). 2021 Dec 29;14(1):151. doi: 10.3390/cancers14010151 (PMC8750310; doi:10.3390/cancers14010151)
Supplement: Supplementary file 1 [file cancers-14-00151-s001.zip › cancers-1526480-supplementary File.pdf]

Supplementary data
Raw Western blots

The images represent the cut membranes that are directly imaged using a phosphoimager.

Figure 1a: Densitometry not performed as not required for the paper – the purpose of the blot is to show expression, i.e. qualitative rather than quantitative

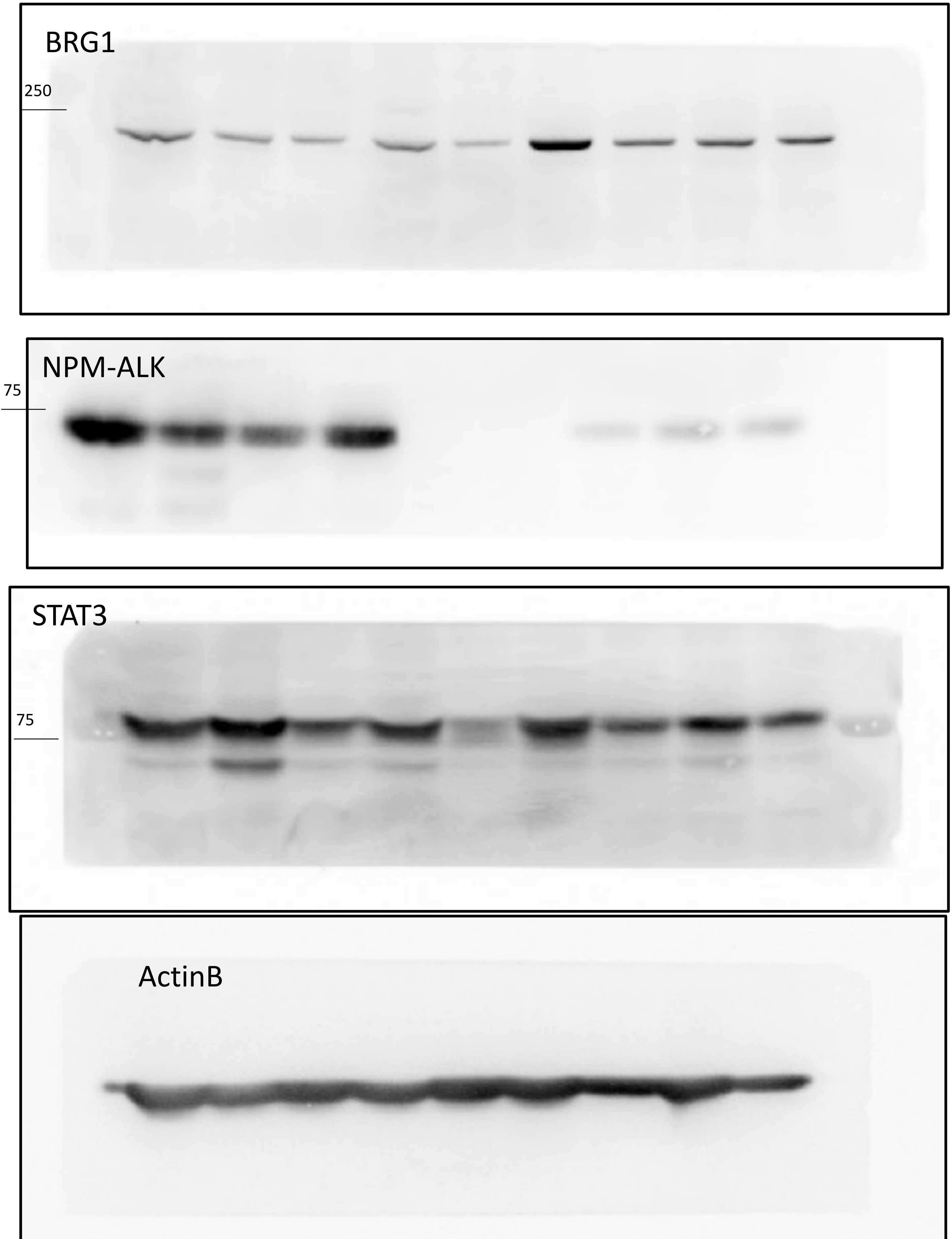

Figure 2a: Numbers below the blots represent the densitometry normalised to tubulin

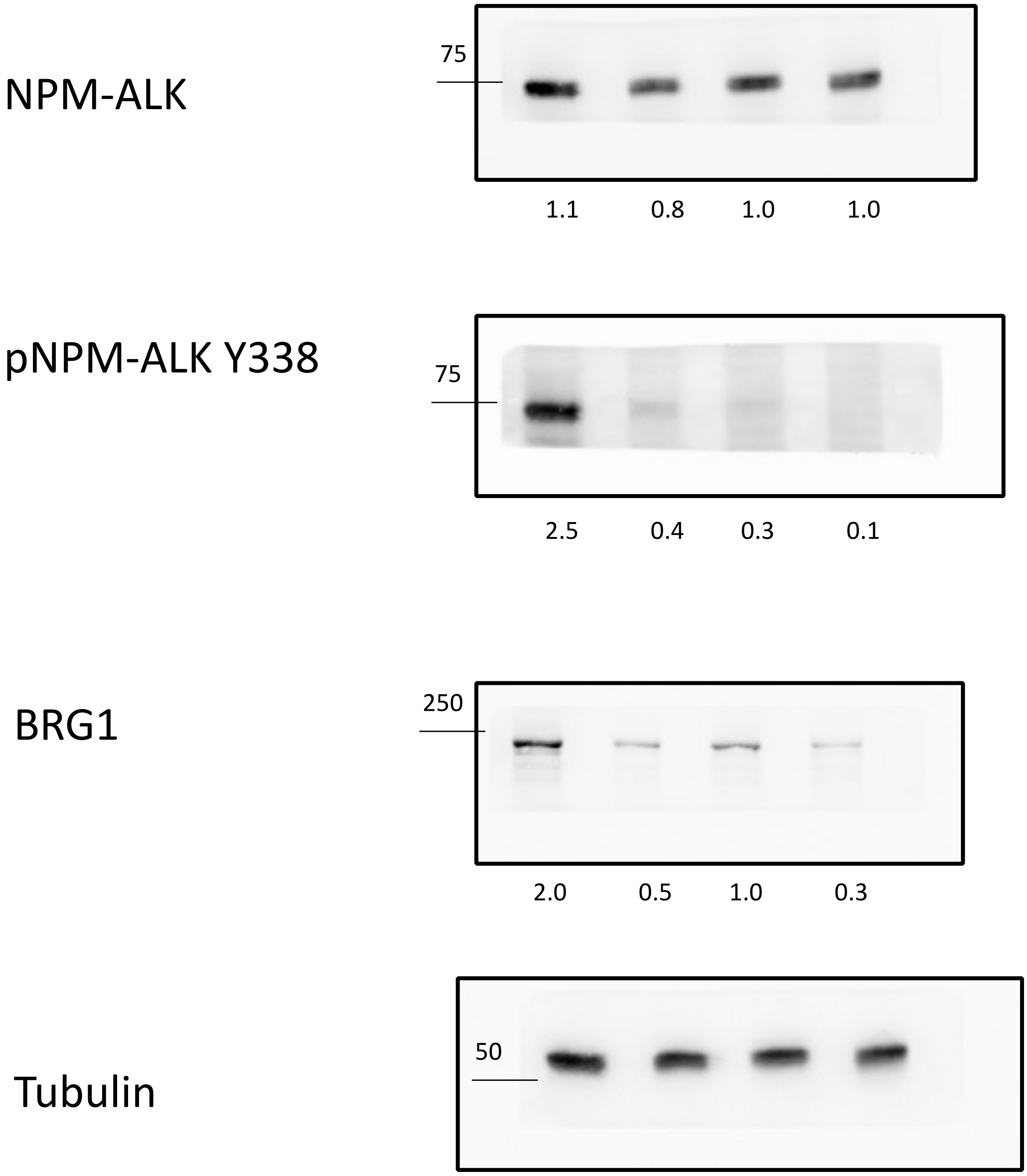

Figure 2b Numbers below the blots represent the densitometry normalised to tubulin . Blot areas to the right indicated by the line were not used in this paper.

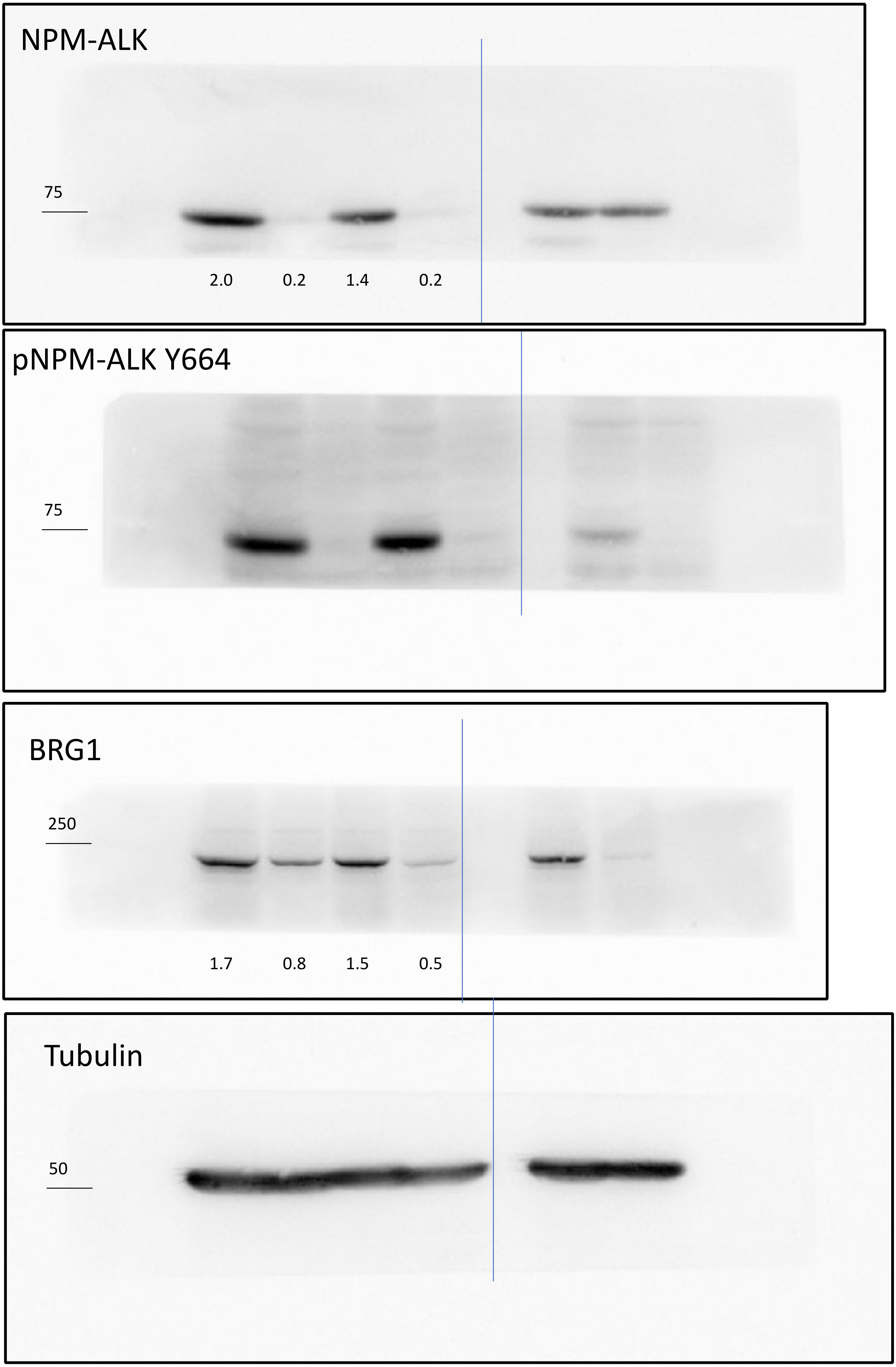

Figure 2c Numbers below the blots represent the densitometry normalised to actin. Blot areas to the right indicated by the line were not used in this paper.

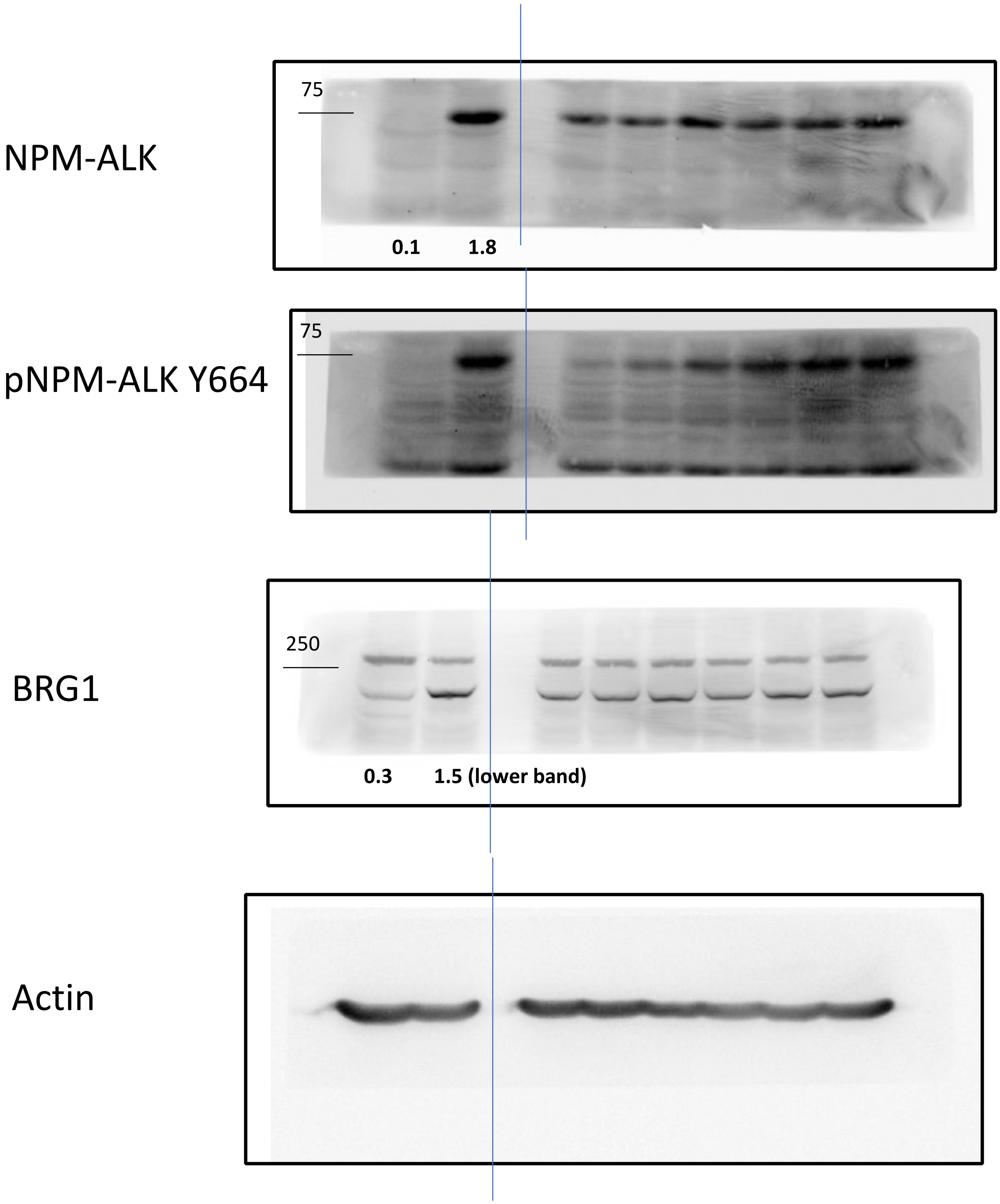

Figure 3C

BRG1

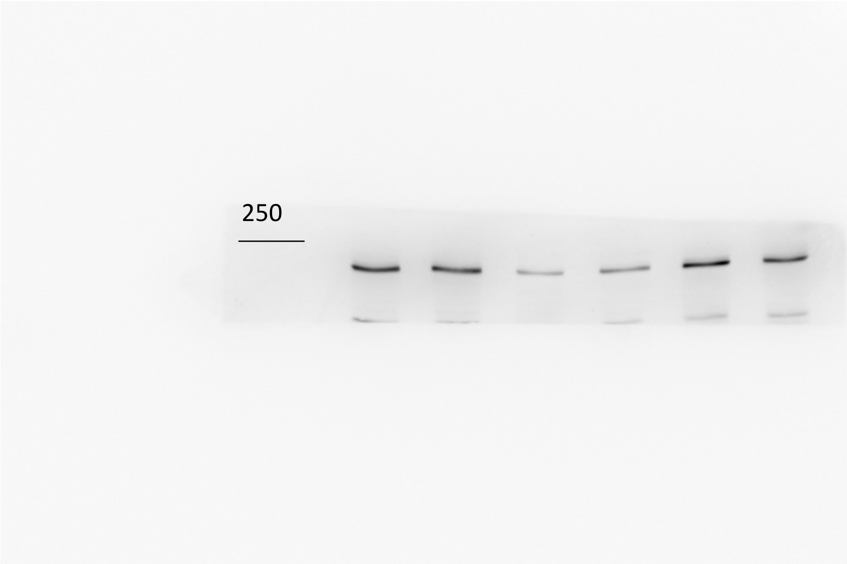

NPM-ALK

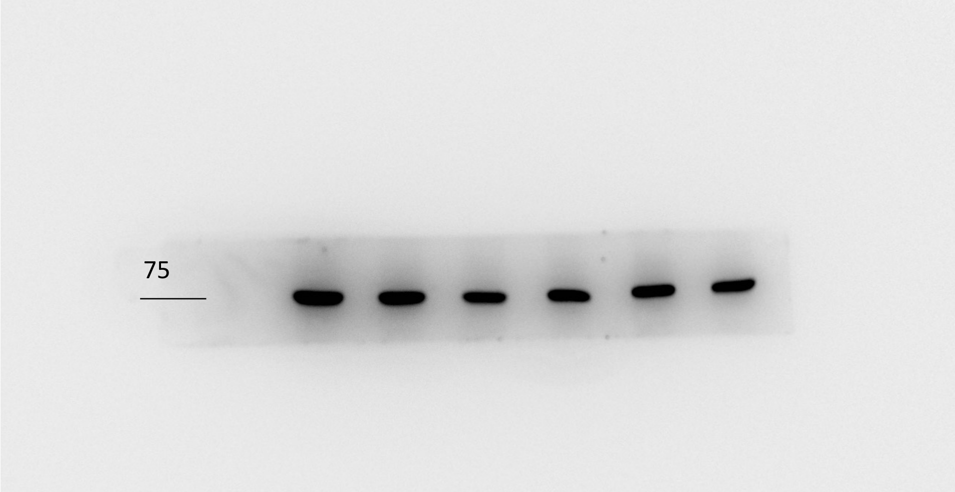

Figure 3C

pNPM-ALK 1278

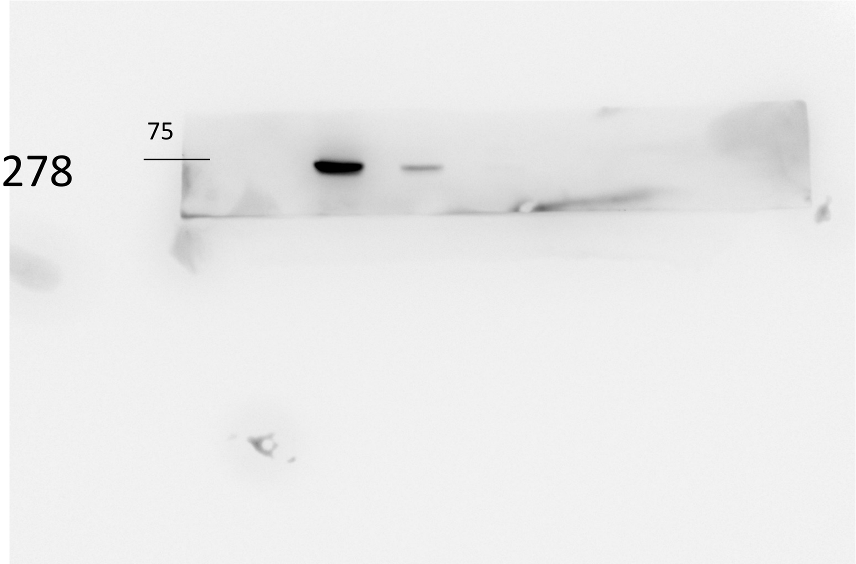

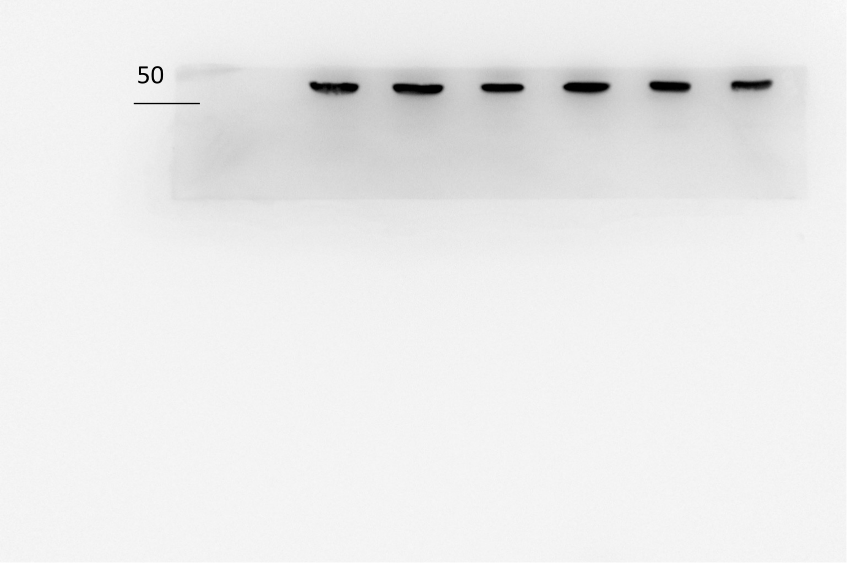

Tubulin
